# Modeling personality antecedents and second language self-efficacy constructs with emerging adults in Japan: Domain-specific matching for assessing global competence in applied contexts

**DOI:** 10.3389/fpsyg.2022.1032573

**Published:** 2022-12-15

**Authors:** Russell Sarwar Kabir, Brandon Kramer, Mayu Koike, Aaron C. Sponseller

**Affiliations:** ^1^Graduate School of Humanities and Social Sciences, Hiroshima University, Higashihiroshima, Japan; ^2^School of Education, Kwansei Gakuin University, Nishinomiya, Japan; ^3^Department of International and English Interdisciplinary Studies, Osaka Jogakuin College, Osaka, Japan

**Keywords:** L2 self-efficacy, intercultural communication, Big Five, foreign language classroom anxiety, L2 listening self-efficacy, L2 speaking self-efficacy

## Abstract

**Introduction:**

Research on self–efficacy in intercultural communication (SEIC) provided validity evidence for second language (L2) self-efficacy domains. However, it lacked (1) an analysis of individual differences in personality as antecedents, (2) divergent validity from anxiety variables (i.e., foreign language classroom anxiety; FLCA), and (3) disambiguation from speaking (S-SE) and listening (L-SE) skill-specific self-efficacy types.

**Methods:**

We conducted structural equation modeling of L2 self-efficacy and anxiety as response variables predicted by the Big Five model of personality in the context of Japanese undergraduate students at three university sites (*n* = 373), and a geographically diverse online survey of emerging adults (*n* = 1,326) throughout Japan.

**Results:**

The final model for the nationally representative sample showed that SEIC was predicted by all identified personality factors. Differentially supported paths were observed linking L-SE with Conscientiousness (β = 0.24) and Extraversion (β = 0.16), and S-SE with Extraversion (β = 0.24) and Neuroticism (β = −0.12). The fear of failure factor of FLCA was predicted positively by Neuroticism (β = 0.25) and, surprisingly, Conscientiousness (β = 0.10), and negatively by Extraversion (β = −0.13). Relationships to Openness to Experience were only supported for SEIC (β = 0.17) and S-SE (β = 0.12).

**Discussion:**

These findings provide specificity matching for personality and L2 self-efficacy domains as empirical advances for assessing global competence within the context of Japan. Implications for cultural influences on self-efficacy and applied educational practices in language and intercultural learning are discussed.

## Introduction

Language and intercultural learning contexts require opportunities for student engagement and interaction as the sources of experiential capital that drive development in “global competence.” Examples of formal contexts to improve this capacity include study abroad, virtual exchange learning, negotiation practice, and designed simulations embedded in structured coursework on intercultural communication. However, global competence is defined in a broad manner, making it difficult to operationalize and track as a measure of student growth. In contrast, personality traits and their measurement frameworks offer strong theoretical grounding and provide a more consistent point of reference across applications with known links to life outcomes. The Big Five personality traits play an important role in the history of second language acquisition (SLA) research as theoretically rich sources of individual differences in the variation of L2 language skills (Dewaele and Furnham, 1999; Dewaele, 2012, 2022; Piechurska-Kuciel, 2020). While personality shapes motivation and anxiety processes, it is often neglected in research applications to SLA (Dewaele, 2012; Piechurska-Kuciel, 2020), possibly due to its higher-order variable status. Operationalizations of self-efficacy (SE), on the other hand, anchor learner beliefs about language and intercultural learning competencies more closely to SLA skills and processes (Irie, 2022). Despite this advantage in specificity, validity evidence for these SE domains is often limited and seldom investigated in tandem with personality factors. To address this gap, we take a model-based approach to understanding the structural relations between personality and applied L2 SE factors.

Given the role that SE plays as a construct relevant to positive psychology and youth development (Shek et al., 2012; Tsang et al., 2012), intercultural effectiveness (e.g., Mendenhall et al., 2008) and related capability-based frameworks (e.g., MacNab and Worthley, 2012), further investigation into contributing factors among adolescents and emerging adults is warranted. Providing specificity matching could: (1) Help practitioners interpret skill-related sources of changes in global competence (e.g., selection and socialization effects from study abroad; Zimmermann and Neyer, 2013; Zimmermann et al., 2021); (2) frame the influence of personality on learning design choices (e.g., toward maximizing the potential of classroom contexts, such as high trait *Extraversion* in the performance of role plays; Karlin and Karlin, 2017); (3) clarify cultural heterogeneity in the dynamic relationship between sources of SE (e.g., socially conveyed sources of SE, Ahn et al., 2016) and anxiety (e.g., fear of failure); (4) allow researchers to adjust for the influence of personality as a source of trait variance in second language (L2)-related SE interventions (e.g., more precise measurement protocols in Sudina (2021), Irie (2022); communicative SE tools, Harris, 2022); and (5) offer clarity about the policy aims to promote “global competence” at both institutions of higher education and in professional development settings as endpoints. Answering this question would require data beyond ordinary university student populations at selected sites and instead examine a panel of geographically diverse individuals within the life stage of emerging adulthood. Described in the following review of the literature, we contend that such an examination would also broaden the findings for specific skill links to learner beliefs to this more expansive group, thereby enhancing our understanding of psychometric properties for localized instruments of personality and L2 SE factors, providing a basis of comparison to student populations, and potentially challenging existing theories of self-enhancement that use broad measurement protocols (e.g., general self-efficacy) in highly represented research samples (i.e., W.E.I.R.D. populations) in the process.

### The broad domain of self-efficacy for global competency

Global competence consists of a broad and multidimensional capacity to possess the intercultural readiness that is prescribed for current students and the workforce alike. According to the (Programme for International Student Assessment [PISA] Report, 2018), Volume VI, global competence is defined in terms of student skills for examining local and global issues, understanding diverse worldviews, engaging in open, appropriate, and effective communication across cultures, taking action for collective wellbeing, and linking the knowledge, skills, and attitudes that are needed to thrive in an interconnected world (Organization for Economic Co-operation and Development [OECD], 2020). Targeted in forecasting of the educational metrics outlook toward 2030, a global competence assessment section was added to the 2018 data cycle of the PISA. Despite the participation of countries like South Korea, Singapore, and Hong Kong throughout Asia, educational institutions in Japan did not incorporate this section for global competence assessment. While reasonable objections to the international indicators-based framework for educational outcomes have been made (Komatsu and Rappleye, 2021), policies toward global competence, such as the *global human resources* initiatives within Japan have been appraised for their features and harmonization (Yonezawa and Shimmi, 2017).

Some differences in this conceptualization of global human resources have been discovered by document analysis (Hofmeyr, 2021), but many components overlap with global competence. The lack of tracked indicators for global competence further contrasts with the ostensible policy commitment to foster and promote “top global” projects at higher education institutions (HEIs) in Japan. The results of the Organization for Economic Co-operation and Development [OECD] (2020) Report indicated that “self-efficacy for global competency” was a major driver of inclusivity, underscoring the need to investigate the ability of students at HEIs to obtain a capacity for global competence and its incumbent factors or precipitating processes. However, a key tension exists between self-efficacy and achievement indicators among youth in the country of Japan. Despite boasting high indicators in domains like literacy and reading performance, Japan’s educational system is characterized by some of the lowest levels of reported general self-efficacy for 15-year-old students at 65%, and most notably, one of the highest rates of fear of failure at 77% (Organization for Economic Co-operation and Development [OECD], 2019). In fact, among all countries in the PISA, 2018 Report, Japan was the only country where a negative relationship between test performance and self-efficacy was observed (Organization for Economic Co-operation and Development [OECD], 2019).

Despite a lack of a clear policy consensus, problem-solving and foreign language skills with a special emphasis on English communication ability, co-occurred the most strongly for the Japanese higher education context (Hofmeyr, 2021), suggesting some priority about curricular components. The role of *English* in Japan’s educational and workforce landscape is debated, with some researchers finding in favor of its facilitating role for globalization processes (e.g., Morita, 2017), and others finding marginal proliferation changes limited to occupational necessity, with a relative dominance of receptive (e.g., listening) to productive (e.g., speaking) skill use in Japanese workplaces (Terasawa, 2021). These contexts represent a connection between the training ground and endpoints of educational programming for emerging adults who will enter the workforce in Japan. However, the outcomes of communicative and global competence are affected by several factors, such as personality (Apple, 2011; Dewaele, 2012; Piechurska-Kuciel, 2020), prior international experience, and aptitude on language proficiency tests as predictor variables. The combination of lower levels of self-efficacy and higher levels of fear of failure writ-large might be considered likely to interact, underlie, or otherwise exacerbate levels of foreign language anxiety and self-efficacy in skills domains such as speaking (S-SE), listening (L-SE), or self-efficacy in intercultural communication (SEIC). Together, these dependent variables might be considered focal constructs that cut across policy aims, HEI classrooms, and other learning experiences that facilitate global competence as a “fundamental competency for working persons” in Japan (Yonezawa and Shimmi, 2017).

### The specific domains of second language self-efficacy

Self-efficacy is framed as a capability and lower-order variable in language learning contexts (Dewaele, 2012), defined as an individual belief’s that they are able to “organize and execute the courses of action required to produce given attainments” (Bandura, 1997; Graham, 2022). Personal agency plays a central role in Bandura’s social cognitive theory and in strategic planning as a phase of self-regulated learning (Graham, 2022). Notably, self-efficacy is anchored to specific domains (Bandura, 2006), as beliefs adhere to a continuum of behaviors bounded by performance constraints (for review: Irie, 2022). For language learning and intercultural competence as domains, criteria include engaging actively in controlled interactions of social significance, observing peers perform in such interactions, seeking and receiving constructive feedback, and overcoming emotional arousal to enhance performance (Mak and Tran, 2001; Li and Gasser, 2005; Kabir and Sponseller, 2020). Related studies of language learners integrating mindset and grit theory (Usher et al., 2019; Khajavy et al., 2021) have revealed some of the properties of self-efficacy and clarified the benefits of a domain-specific approach in other studies (e.g., L2 grit; Sudina and Plonsky, 2021; Teimouri et al., 2021). Scholars have suggested that generating a breadth of experiential capital leads to gains in L2 self-efficacy (Irie and Brewster, 2014).

Skilled receptivity and productivity in communication are relevant to L2 language learning and valued in training contexts for communicative competence. Despite cross-cutting evidence in favor of its role as a biomarker of social behavior, the listening process has been described as more difficult to monitor and assess than some of the other four skills. Specifically, listeners must parse chunks of speech in a sequence that leads to semantic integration, usually in the form of vocabulary items as learned units that connect to phrases (Hu and Jiang, 2011). In studies of listening as a naturalistic stimulus among humans, listening to stories resulted in highly reproducible cortical responses that are thought to reflect the encoding of semantics, inference to concepts, and the relating of concepts to one another (Zhang et al., 2020). Listening in an L2 is uniquely effortful and slow, imposing heavy demands on working memory in studies of cross-modal priming (Wu and Ma, 2016), but forming an important skill objective in pedagogical processes (e.g., connected speech, Hahn, 2018). Speaking is a key domain for interactional productivity as it requires sufficient coordination to result in illocutionary force. Developmental processes for vocal production have been posited for adult-like vocalizations and the mapping of new forms onto lexical representations (Vihman, 2022). In addition, vocal reproduction through elicited imitation tests have emerged as reconstructive, integrative, modality independent, and indirectly communicative sources of variation in global proficiency (Wu et al., 2021). Together, empirical work suggests that critical examinations of self-efficacy take listening and speaking skills into account as unique sources of domain-specificity that reflect key language and learning processes.

Bringing those skills into practice, intercultural learning is a source of sociolinguistic and pragmatic awareness (McConachy and Spencer-Oatey, 2020), and is made available to learners in contexts like study abroad (Varela, 2017) and virtual exchange learning (Wickline et al., 2021). In one key study, performance on a modified Cloze test was used to determine L2 proficiency gains during 3 months of study abroad and analyzed with a comprehensive account of individual differences, which further clarified a refined focus on self-efficacy and anxiety (Hessel, 2017). This study provided insights into the psychological factors of language learners who have traveled as a form of committed behavior to personal growth. Delineating sources of variability, the results of multiple regression analysis in the study suggested that proficiency gain from 3 months abroad could be associated to three key factors: L2 self-efficacy, L2 use anxiety (construed as facilitating anxiety), and attitudes toward one’s own national group. The findings suggested that individual differences in factors for using the L2 in social interactions can predict degrees of proficiency gain (Hessel, 2017). Overall, the study underscored the need to explore the linguistic affordances that learners have or seek in *interactions* with other L2 learners. This includes the classroom, as experiential teaching of intercultural competence facilitates interaction (Spencer-Oatey and Franklin, 2009; Kemmelmeier and Kusano, 2018).

### The dynamic and culturally shaped interplay of second language self-efficacy and anxiety

Experiences with failure or apprehension and related anxiety due to language performance and communication would result in lower levels of self-views like L2 learner beliefs. Studies have shown a dynamic interplay between SE and anxiety due to their facilitating and debilitating state-like components. In the case of SE, if one relies too much on their self-efficacious beliefs, it might lead to ambivalence or even overconfidence (Usher et al., 2019). For foreign language anxiety, fear of failure and communication apprehension are psychometrically identified factors in research on the psychology of language learning. Work on foreign language anxiety reduction has been explored and compared with various formats (for review: Toyama and Yamazaki, 2021), however, young adults in Japan exhibit and experience communication barriers from cultural expectations (e.g., Kowner, 2004) like the effects of silence in educational contexts (Harumi, 2011; Sasaki and Ortlieb, 2017; Yashima et al., 2018), that would place constraints upon the depth of interactions requisite for language learning and productive skills development at HEIs. While another study by Toyama and Yamazaki (2018) examined the psychometric properties of the Japanese version of the Foreign Language Classroom Anxiety Scale (FLCAS), the factors for fear of failure and communication apprehension have yet to be investigated for their interactions with L2 self-efficacy constructs or personality traits in Japan. The dynamic interplay and cultural factors suggest that an optimum exists for learning and performance in communication as a learned skill or set of skills and might interact with or contribute to upstream effects in emerging adulthood (e.g., *via* personality development), though the intersection between trait and state measures of motivational impact remains theoretically disconnected (Dörnyei, 2014).

### Personality factors as antecedents to second language self-efficacy and anxiety

Variability in personality, language, and intercultural competence would be expected to be interrelated, as they rely on a common substrate for the ability to engage in and be effective at intercultural communication. Illustrating these connections, a large meta-analytic study of 10,672 individuals found that cross-cultural self-efficacy was moderately correlated with sociocultural adaptation at a magnitude greater than the correlations for personality variables from the Big Five (Wilson et al., 2013). According to trait theory, some indicators for the more domain-specific SEIC would be expected to depend upon a profile of dispositional characteristics for interpersonal engagement (e.g., *Openness to Experience*, *Extraversion*) and achievement motivation (e.g., *Conscientiousness*) within a web that includes a broader capability to relate and interact effectively in intercultural settings. S-SE and L-SE would require similar tendencies to try to engage in situated interactional contexts in an L2 (e.g., *Extraversion, Openness to Experience*), and manage trait-like forms of anxiety (e.g., *Neuroticism, Fear of Failure, Communication Apprehension*) to perform effectively, receive feedback, and sustain the motivation to improve (e.g., *Conscientiousness*). A 5-year follow-up study of personality suggested effects for *Openness*, *Agreeableness*, and *Neuroticism* as sojourn-induced contextual changes, which occurred early after sojourn, and sojourn effects on *Openness* and *Neuroticism* were shown to sustain longitudinally (Richter et al., 2020). In this way, prior studies have specifically suggested that international experience and personality are sources of individual differences that potentially influence the dynamic of self-efficacy for global competency and forms of anxiety.

In sum, a gap remains to structurally address the relations between individual differences in personality, foreign language anxiety, and self-efficacy in contexts marked by factors of cultural heterogeneity in educational management and commitment to global competence, such as Japan. As detailed above, applications and measurement tools of self-efficacy are stipulated under domain-specific, as opposed to domain-general, conditions, and factors of personality as higher-order variables might predict forms of L2 SE and anxiety.

### The present study

Our previous study discovered correlations between SEIC and two factors of intercultural effectiveness, *Interpersonal Engagement* and *Continuous Learning*, suggesting trait variance for similar constructs like *Extraversion* and *Conscientiousness*, respectively (Kabir and Sponseller, 2020). However, we did not disambiguate it from personality traits as higher order dispositional characteristics, which would bolster its construct representation as a lower-order characteristic adaptation, as well as connect to broader frameworks like the Big Five. This is a key test because lower-order positive psychology constructs have been tested for L2 self-efficacy with 212 university students in Japan (Lake, 2013, 2016), finding the mid-level to be most appropriate, and L2 self-efficacy variables for language skills domains have been shown to correlate with positive self-views in Taiwan, the United States, and Japan, observing lower mean levels of L2 self-efficacy in Japan (Chen et al., 2020). We extend this test with personality antecedents to L-SE, S-SE, and possible contributions of foreign language classroom anxiety, surmising that the relationships would be situated within the context of applications and scrutinized under constraints by other factors (e.g., prior international experience) statistically testable with a model fitting and selection process. Structural equation modeling (SEM) was chosen due to its strengths for simultaneously estimating these variable relations in a principled manner.

### Purpose of the study

Building on these findings, this study carefully models personality traits as antecedents to the specific domains of SEIC, S-SE, L-SE, and FLCA, using the *lavaan* package in R (Rosseel, 2012). The standardized coefficients from the structural model were expected to clarify the pattern(s) of traits that might relate to this capability for both university students and emerging adults (aged 18–29) in Japan as a source of construct validity and psychometric properties, as well as provide insights on the relationship(s) between anxiety and self-efficacy that might be related to global competence.

*Research Question 1:* Do personality traits relate to L2 self-efficacy constructs in a geographically diverse sample of emerging adults in Japan?

Our first goal for this project was to investigate the relationship between the Big Five model and an individual’s belief in their ability to interact effectively in intercultural communication situations while using an L2 (English). Adjusting for covariates, we aimed to build upon the validity evidence of an instrument to measure SEIC (Kabir and Sponseller, 2020). Specifically, we expected that relationships for *Extraversion* and *Conscientiousness* and SEIC that were observed in a modeling of the same constructs in a university sample from three sites (Supplementary material 1) would generalize to a larger sample of emerging adults and represent a form of self-efficacy for global competency. Previous research suggests that self-efficacy should broadly and moderately oppose anxiety-related constructs, therefore, we expected the following hypothesized relationships.

H1: Standardized coefficients for SEIC will support positive (negative) relationships to Extraversion and Conscientiousness (Neuroticism), but not Openness to Experience.

In a meta-analysis by Shirvan et al. (2019), language anxiety, motivation, and perceived communicative competence were moderate correlates of willingness to communicate (WTC; MacIntyre et al., 1998). International posture, a related construct specified by Yashima (2002, 2009) was explored in a previous study with personality traits, finding in favor of *Openness to Experience* and *Extraversion* in one structural equation model (Toyama and Yamazaki, 2020). Due to prior relationships and validation approaches related to WTC measures and self- and task-oriented foreign language listening anxiety, for the two skill-specific SE types, we expected that L-SE and S-SE would be related to *Extraversion* and *Openness to Experience* (and oppose the direction of Neuroticism).

H2: Standardized coefficients for L-SE and S-SE will support positive (negative) relationships to Extraversion and Openness to Experience (Neuroticism).

*Research Question 2:* Do the antecedents of personality relate to FLCA factors in a geographically sample of emerging adults in Japan?

From a theory-driven perspective of facets organized by Piechurska-Kuciel (2020) and a similar nomological network by Ang and Van Dyne (2008), the FLCA factors of *communication apprehension* (CA) and *fear of failure* (FOF) in classroom contexts would be predicted by trait variance in personality. We expected that cross-sectional observations from a web survey research panel of emerging adults would indicate negative relationships to all factors except Neuroticism, which would be positive.

H3: Standardized coefficients for FLCA-CA and FLCA-FOF will support negative (positive) relationships to Extraversion, Conscientiousness, and Openness to Experience (Neuroticism).

## Materials and methods

A comprehensive set of demographic items and psychometric instruments was given in Japanese language to university students and emerging adults as research participants. The first survey built upon the findings of the SEIC validation (Kabir and Sponseller, 2020) with an extended collection of the same test battery for SEIC and personality traits. The survey data was collected from three university sites in Japan (*n* = 373) and employed purposive and convenience-based sampling approaches. This data served as the training set for the SEM relations between personality and SEIC adjusting for the influence of gender as a form of partial secondary data analysis (Supplementary material 1). *Extraversion* and *Conscientiousness* showed statistically supported paths to SEIC. As that prior study used a non-probability-based sampling strategy, we chose to append it as Supplementary material, but use the findings as a basis for hypothesis formation and model scrutiny (see section “Discussion”), as well as the foundational assumptions for pre-registration with a nationally representative sample. The survey items for the study variables are also available (Supplementary material 2).

Narrowing our scope and inferences to the second survey with a larger pool of participants, our study focuses on the results of the web research panel that employed randomized recruitment of geographically diverse respondents throughout Japan with specific age screening criteria to the span of emerging adults. Sample size justification was carried out based on resource constraints for a web research panel (Lakens, 2022), and a sensitivity analysis was conducted to identify effect sizes that the sample could detect with 80% power (Wang and Rhemtulla, 2021). The plan for the study was pre-registered prior to analysis on the Open Science Framework (see section “Data availability statement”).

### Study participants

A panel of 1,364 emerging adults (661 males, 692 females, 8 non-binary and gender-diverse) responded to an online survey built and distributed with the Qualtrics research platform in an observational study design. Participants were recruited with randomized sampling techniques with the assistance of a research coordinator who followed inclusion and exclusion criteria for the span of emerging adults aged 18–29 in Japan and range of locations per the population density of Japan by prefecture. Participants reported currently residing in one of the 47 prefectures in Japan, indicating geographic diversity for the sample. All respondents were L1 users of Japanese and of Japanese nationality. During data cleaning, age-related data was missing from 3 participants and was considered missing completely at random. The age range of the full sample was 18–29, with a mean age of 25.22, standard deviation of 3.08, and median age of 26.

### Measures

#### Demographic variables

Age, gender, university student status, major, native language, prefecture of residence, marital and occupational status, ethnicity, and socioeconomic status (subjective SES; Nakashima and Yanagisawa, 2015; objective SES as highest parental and self-related educational attainment) were collected in the nationally representative online survey. Chi-square tests and exploratory sub-group analyses are planned for future research, the present study focuses on planned confirmatory analysis using university and non-university student status. Due to the lack of an effect for gender in the first modeling procedure with university students (Supplementary material 1), and the focus on extending beyond the age range of university students and site-specific sampling to a broader group of emerging adults, age was the only demographic variable entered into SEM.

#### International experience

Responses to international experience questions were collected in reference to Takeuchi et al. (2005) and Ott and Iskhakova (2019), with three response options with related item survey logic: whether respondent has previously traveled abroad, number of countries visited in their lifetime, and total length of time spent abroad (i.e., with example demarcations in days, weeks, months, and/or years). For the sake of simplicity, international experience was treated as a binary exogenous variable using the first yes-or-no item, “Have you ever traveled abroad?,” dummy-coded for the present modeling procedure.

#### English language aptitude test experience

Prior experience with language proficiency exams in English was assessed with the question, “Which of the following tests have you taken most recently?” and choices of, “TOEIC, TOEFL, G-TEC, IELTS, never taken one of the above,” with subsequent display logic for self-reporting scores for the selected tests or informing about another kind of test taken. To simplify the modeling procedure, responses were collapsed into a binary exogenous variable for yes-or-no prior experience.

#### Revised Big Five Markers—Japanese (Apple, 2011)

The Revised Big Five factor markers—Japanese (RB5-J) is a 37-item instrument for assessing personality based on Goldberg’s items that were translated into Japanese and validated with 1,081 students from 12 universities in Japan. Sample items included, “Start conversations,” and “Have a vivid imagination.” In response to the statement, “This personality trait describes me,” participants answered on a scale ranging from 1 to 6. All points on the scale were semantically labeled from 1 (*Strongly Disagree*) to 6 (*Strongly Agree*) in the online table matrix table configuration.

#### Self-efficacy in intercultural communication—Japanese (Peterson et al., 2011; Japanese version by Kabir and Sponseller, 2020)

The SEIC-SF is an 8-item measure that summarizes a domain of self-efficacy as it relates to intercultural communication (e.g., sample item, “How well can you communicate in impromptu situations?”). Previous research on the SEIC-SF by Kabir and Sponseller (2020) extended the original work of Peterson et al. (2011) by establishing cross-cultural validity and measurement invariance across samples of sojourning English teachers in Japan, Japanese teachers of English, and Japanese university students. A six-point Likert scale ranging from 1 (“*I definitely cannot do this*”) to 6 (“*I can do this very well*”) was used, with all points on the scale semantically labeled. Instructions stipulated that the competency is anchored to one’s ability to communicate in English as the L2 for self-assessment.

#### Second language speaking self-efficacy (Hicks and McLean, 2014)

The S-SE is a 20-item instrument developed with Japanese university students. Technical item quality was evaluated with Rasch principal components analysis, and nomothetic span was investigated against the WTC model and constructs in its validation. In addition, external validity was previously investigated and supported for the items to discriminate from foreign language speaking anxiety. Items include can-do statements such as “I can respond in English to greetings from international students on campus.” Participants responded to a six-point Likert scale ranging from 1 (“*I definitely cannot do it*”) to 6 (“*I can definitely do it*”). Each point on the scale was semantically labeled.

#### Second language listening self-efficacy (Kramer and Denison, 2016)

The L-SE is an L2 domain-specific instrument of 14 items drawn from Burrows (2013) and fitted with Bandura (2006) prescriptions for self-efficacy scale development. The scale was validated for Japanese EFL learners achieving elements of content relevance from interview data, technical item quality from Rasch rating scale modeling, convergent validity through moderately positive correlations with vocabulary knowledge and divergent validity through moderately negative correlations with foreign language listening anxiety. Sample items included “If I heard an English conversation at the level of a junior high school textbook, I would understand it,” and “If I watched the news in English, I would understand it.” A six-point Likert scale ranging from 1 (“*I definitely cannot do it*”) to 6 (“*I can definitely do it*”) was used. Each point on the scale was semantically labeled.

#### Foreign language classroom anxiety scale— Japanese (Horwitz et al., 1986; Japanese version by Yashima et al., 2009; Toyama and Yamazaki, 2018)

The FLCAS is a 33-item instrument translated into Japanese and back-translated into English, with instructions for responding to the statements adapted to specify English as the foreign language for student self-assessment (e.g., “It frightens me when I don’t understand what the teacher is saying in English”). In accordance with the prior validation, a 5-point Likert scale from 1 (*Strongly Disagree*) to 5 (*Strongly Agree*) was used. Each point on the scale was semantically labeled.

### Procedures

The rationale for inclusion criteria was based on screening criteria for individuals within the age range of young and emerging adults and whose first language was Japanese. All respondents fit these criteria. Additionally, data cleaning prioritized complete item level data and low average long-string responses. The *careless* package in R was used to determine potential careless or insufficient effort responding patterns for further inspection (Yentes and Wilhelm, 2021). As a part of data cleaning, data belonging to 39 participants were shown to have more than 5 average long-string patterns, indicating a plausible tendency for “straightlining” that we judged as a form of careless response. These participants were deleted listwise from the dataset.

Complete cases were then re-checked by summing complete cases with the *psych* package (analytic sample size, *N* = 1,326). For the analytic sample, indicator coding for university status revealed that data from current university students (*n* = 218, *M*_*age*_ = 21.14, *SD* = 2.37, median age = 21) and non-university students (*n* = 1,108, *M*_*age*_ = 26.01, *SD* = 2.53, median age = 27) within the age range of emerging adults were collected. Outside of the three missing data points for age, there were no cases of incomplete data, and indicator variables (i.e., dummy coding) for current university status, international experience (“yes” to travel abroad; *n* = 600; “no” to travel abroad, *n* = 726), and language aptitude experience (“yes” to proficiency test taken before, *n* = 497; “no” *n* = 829) were prepared for analysis.

The protocol of the study project was approved by the ethical research committee at Hiroshima University. Participants were provided with information about the content of the questionnaire and the purpose of the study at the beginning of the survey. All respondents gave their informed consent to participate at the start of the survey and allowed the use of their data for analysis.

### Analytical plan

To assess the pattern of responses of the study variables, descriptive analyses were performed. Items were treated as continuous and multiple forms of reliability were estimated (Dunn et al., 2014). Confirmatory factor analysis (CFA), which is often chosen to provide measurement models that systematically examine the structural validity of latent constructs, was used to confirm the factor structures of the constructs and their measurement invariance for further analysis (Brown, 2015). Finally, structural equation modeling (SEM) was conducted to simultaneously estimate and examine the relations between the factors (Hu and Bentler, 1999; Kline, 2011). Several indices of model fit were considered, namely the Chi-square (χ^2^), Comparative Fit Index (CFI), Tucker-Lewis Index (TLI), Standardized Root Mean Square Residual (SRMR), and Root Mean Square Error of Approximation (RMSEA), in line with quantitative research reporting standards (Appelbaum et al., 2018).

## Results

### Descriptive statistics and reliability analysis

The results of the descriptive analyses are summarized in Table 1. Estimates for the study variables among the truncated sample of the 1,326 emerging adults were calculated in *R* (Version 4.1.2, R Core Team, 2021). As depicted in Table 1, Cronbach’s α (*psychometric* package) and McDonald’s ω (*MBESS* package) values were estimated and found to exceed 0.70 for all study variables, in keeping with conventional guidelines for supporting factor reliability (Dunn et al., 2014). Correlational analysis of the study variables is also provided in Table 2.

**TABLE 1:** Descriptive statistics and reliability for the study variables with emerging adults (*n* = 1,326) in Japan.

Study variable	Emerging adults in Japan
**Revised Big Five factor markers (*M*, *SD*)**	
Neuroticism [4 items; α = 0.81, ω = 0.81 (0.79, 0.83)]	4.12 (1.07)
Conscientiousness [3 items; α = 0.75, ω = 0.75 (0.72, 0.77)]	3.51 (1.02)
Extraversion [4 items; α = 0.87, ω = 0.87 (0.85, 0.88)]	2.98 (1.21)
Openness to experience [3 items; α = 0.74, ω = 0.77 (0.75, 0.80)]	3.56 (1.07)
**Self-efficacy in intercultural communication (*M*, *SD*)**	
SEIC [8 items; α = 0.95, ω = 0.95 (0.94, 0.95)]	2.46 (1.11)
**Speaking self-efficacy (*M*, *SD*)**	
S-SE [20 items; α = 0.98, ω = 0.98 (0.98, 0.98)]	2.38 (1.17)
**Listening self-efficacy (*M*, *SD*)**	
L-SE [5 items; α = 0.92, ω = 0.93 (0.92, 0.93)]	2.99 (1.20)
**Foreign language classroom anxiety (*M*, *SD*)**	
Communication apprehension [7 items; α = 0.79, ω = 0.83 (0.82, 0.85)]	3.07 (0.80)
Fear of failure [5 items; α = 0.83, ω = 0.83 (0.81, 0.85)]	3.17 (0.91)

*M* and *SD* are used to represent mean and standard deviation, respectively. α and ω are used to represent Cronbach’s alpha and McDonald’s omega coefficients, respectively. Confidence intervals [95% CI] and standard errors were estimated for McDonald’s omega with the bias-corrected and accelerated bootstrap set to 5,000 replications. Response categories for the RB5, SEIC, S-SE, and L-SE ranged from 1 to 6, and FLCAS ranged from 1 to 5.

**TABLE 2:** Model comparison of factor structures and psychometric properties for the study instruments (*n* = 1,326).

Model		*df*	Minimum function test statistic (χ^2^)	χ^2^ *P*-value	CFI	TLI	SRMR	RMSEA (CI)
Revised Big Five factor markers	1-factor model (37-item)	629	7794.986	0.000	0.609	0.586	0.112	0.093 (0.091–0.094)
	5-factor model (35-item)	550	3474.494	0.000	0.831	0.817	0.065	0.063 (0.062–0.065)
	4-factor model (14-item)*	71	396.234	0.000	0.940	0.923	0.056	0.059 (0.054–0.064)
Self-efficacy in intercultural communication	1-factor model* (8-item)	20	213.308	0.000	0.955	0.937	0.029	0.085 (0.078–0.093)
L2 Speaking self-efficacy	1-factor model (20-item)	170	1539.997	0.000	0.910	0.899	0.035	0.078 (0.076–0.080)
	1-factor model (20-item with residual covariances)*	166	1123.611	0.000	0.937	0.928	0.031	0.066 (0.064–0.068)
L2 Listening self-efficacy	1-factor model (14-item)	77	3495.484	0.000	0.722	0.672	0.124	0.183 (0.179–0.187)
	1-factor model (5-item)*	5	54.854	0.000	0.983	0.966	0.021	0.087 (0.070–0.105)
Foreign language classroom anxiety	2-factor model* (12-item)	53	320.797	0.000	0.949	0.937	0.035	0.062 (0.056–0.067)

The values for the test and fit statistics are reported for the results of models with the robust maximum likelihood estimator. CFI, Comparative Fit Index; TLI, Tucker-Lewis Index; SRMR, Standardized Root Mean Square Residual; RMSEA, Root Mean Square Error of Approximation; RMSEA values refer to results scaled with the Yuan-Bentler correction factor.

*Denotes accepted factor models.

### Confirmatory factor analysis

Structural validity was evaluated using the *lavaan* package in *R* (Rosseel, 2012; version 0.6–7, Rosseel et al., 2020). The robust maximum likelihood estimator was used, and model selection involved the comparison of fit indices and information criteria. Model fit for CFA and SEM was assessed in terms of Squared Root-Mean Square Residual (SRMR; acceptable < 0.08), the Tucker Lewis Index (TLI; acceptable > 0.90), the Root Mean Square Error of Approximation (RMSEA; acceptable < 0.08), and the Comparative Fit Index (CFI; acceptable > 0.90, Hooper et al., 2008). Latent factors were scaled by fixing first factor loadings.

Measurement invariance testing was also performed using the *lavaan* package with special attention to the status of participants as emerging adults (*N* = 1,326) who were currently attending university (*n* = 218, *M*_*age*_ = 21.14, *SD* = 2.37, median age = 21) and other emerging adults who were not (*n* = 1,108, *M*_*age*_ = 26.01, *SD* = 2.53, median age = 27). The models specified for each of the relevant study variables were checked with likelihood ratio tests for latent variables using the scaled Chi-squared difference test (Satorra and Bentler, 2001).

### The four-factor model of the revised Big Five markers—Japanese

Procedures for model estimation and selection of the RB5-J were conducted. As depicted in Table 2, initial CFA of the items in a 1-factor model indicated poor model fit: Robust χ^2^ (629) = 7794.986, *p* = 0.000; CFI = 0.609; TLI = 0.586; SRMR = 0.112; RMSEA = 0.093 (95% *CI*:0.091–0.094). As observed in the original validation by Apple (2011), the 5-factor model also indicated poor model fit: Robust χ^2^ (550) = 3474.494, *p* = 0.000; CFI = 0.831, TLI = 0.817; SRMR = 0.065; RMSEA = 0.063 (95% *CI*:0.062–0.065), in favor of a 4-factor model: Robust χ*2* (71) = 396.234, *p* = 0.000; CFI = 0.940; TLI = 0.923; SRMR = 0.056; RMSEA = 0.059 (95% *CI*:0.054–0.064). The best fitting 4-factor model was supported, notably with no specification of *Agreeableness* as a factor but with one item assigned to Extraversion instead. Factor loadings ranged from 0.53 to 0.89, with no low loadings. Inspection of the latent variable covariances suggested separation of the factors. The CFA results were comparable to the original calibration of the RB5-J [Apple, 2011; CFI = 0.95; SRMR = 0.05; RMSEA = 0.05 (0.043–0.063)] and validation sample with Japanese EFL students [Apple, 2011; CFI = 0.91; SRMR = 0.07; RMSEA = 0.08 (0.067–0.085)].

Group testing was performed for the current university status indicator. Measurement invariance was tested for configural [χ*2* (142) = 626.61, AIC = 56,675; BIC = 57,173], weak [χ*2* (152) = 640.95, AIC = 56,669; BIC = 57,116; χ*2* difference (10) = 12.713, *p* = 0.24] and strong [χ*2* (162) = 657.75, AIC = 56,666; BIC = 57,061; χ*2* difference (10) = 16.675, *p* = 0.08] level constraints. Information criteria values were lowest for the strong invariance check, supporting a comparison of means between the two groups for the RB5-J.

### One-factor model of self-efficacy in intercultural communication

Table 2 also presents the results for the SEIC-SF-J. Initial CFA of the 1-factor model indicated good model fit: Robust χ*2* (20) = 213.308, *p* = 0.000; CFI = 0.955; TLI = 0.937; SRMR = 0.029; RMSEA = 0.085 (95% *CI*:0.078–0.093). Standardized estimates for the factor loadings ranged from 76 to 87. Measurement invariance for current (non-)university students was tested for configural [χ*2* (40) = 460.70, AIC = 26,755; BIC = 27,004], weak [χ*2* (47) = 466.10, AIC = 26,747; BIC = 26,959; χ*2* difference (7) = 6.5075, *p* = 0.48] and strong [χ*2* (54) = 475.24, AIC = 26,742; BIC = 26,918; χ*2* difference (7) = 9.0454, *p* = 0.25] constraints. Information criteria values were lowest for the strong invariance check, supporting a comparison of means between the two groups for the SEIC-SF-J.

### One-factor model of speaking self-efficacy

For S-SE-J, two models were estimated, as shown in Table 2. Estimation of the 1-factor model indicated adequate fit: Robust χ*2* (170) = 1539.997, *p* = 0.000; CFI = 0.910; TLI = 0.899; SRMR = 0.035; RMSEA = 0.078 (95% *CI*:0.076–0.080). Modification indices indicated substantial overlap for items 1 and 2, 4 and 5, 17 and 18, and 18 and 19. A model estimated with residual correlations for these item-pairs showed improved model fit: Robust χ*2* (167) = 1123.611, *p* = 0.000; CFI = 0.937; TLI = 0.928; SRMR = 0.031; RMSEA = 0.066 (95% *CI*:0.064–0.068). Factor loadings ranged from 0.72 to 0.89. This model was used for subsequent SEM analysis.

Measurement invariance for current (non-)university students was tested for configural [χ^2^ (340) = 3732.9, AIC = 60,263; BIC = 60,886], weak [χ^2^ (359) = 3779.8, AIC = 60,272; BIC = 60,796; χ^2^ difference (19) = 57.994, *p* < 0.001] and strong [χ^2^ (378) = 3838.5, AIC = 60,293; BIC = 60,718; χ^2^ difference (19) = 57.249, *p* < 0.001] constraints. Information criteria values were lowest for the configural invariance check, supporting content but not metric or scalar comparisons for the L2 SE-SE-J and (non-)university students.

### One-factor model of listening self-efficacy

Table 2 presents the results for L-SE-J. Initial CFA of the 14-item 1-factor model indicated poor model fit: Robust χ^2^ (77) = 3495.484, *p* = 0.000; CFI = 0.722; TLI = 0.672; SRMR = 0.124; RMSEA = 0.183 (95% *CI*:0.179–0.187]. We conducted a new measurement model with one of the scale developers (B.K.) for the factor analysis. The co-authors consulted about an item reduction process informed by the theory of self-efficacy, comparisons with item performance in the original Rasch validation, preserving that validation approach to emphasizing a range of item difficulty, and data-driven insights from modification indices. Discussion revealed a double-barreled item for about test-taking (“Center Test or TOEIC”), which was removed. Furthermore, informed by their strongly correlated residuals likely reflecting multi-modal contributions to the listening process, items about listening to Japanese speakers of English, having a conversation over the phone, and listening with subtitles were also removed from the model. Together, re-examination of the item content suggested that items 1, 2, 4, 8, 9, 10, 12, 13, and 14 be removed (see section “Discussion”). A subsequent modeling procedure for a 1-factor model with five items (3, 5, 6, 7, 11) indicated greatly improved model fit: Robust χ^2^ (5) = 54.854, *p* = 0.000; CFI = 0.983; TLI = 0.966; SRMR = 0.021; RMSEA = 0.087 (95% *CI*:0.070–0.105). Factor loadings ranged from 0.63 to 0.92.

Measurement invariance for current (non-)university students was tested for configural [χ^2^ (10) = 78.695, AIC = 17,672; BIC = 17,828], weak [χ^2^ (14) = 85.062, AIC = 17,671; BIC = 17,806; χ^2^ difference (4) = 8.293, *p* = 0.08] and strong [χ^2^ (18) = 86.531, AIC = 17,664; BIC = 17,778; χ^2^ difference (4) = 1.443, *p* = 0.84] constraints. Information criteria values were lowest for the scalar invariance check, supporting mean-based comparisons for the L2 SE-J between university and non-university student emerging adults.

### Two-factor model of the foreign language classroom anxiety scale

The FLCAS was tested for its psychometric properties in accordance with the model put forth by Toyama and Yamazaki (2018). Initial CFA of the 2-factor model indicated good model fit: Robust χ*2* (53) = 320.797, *p* = 0.000; CFI = 0.949; TLI = 0.937; SRMR = 0.035; RMSEA = 0.062 (95% *CI*:0.056–0.067). Factor loadings ranged from 0.60 to 0.80, with one low loading at 0.22 for item 2. Group testing was not performed for the current university status indicator for this model as FLCAS as a classroom variable was expected to differ between groups.

### Structural equation modeling

Structural equation modeling was carried out in *lavaan* to examine the relations between the best-performing factor models of the study variables. As strong measurement invariance was not upheld for some of the study variables (i.e., S-SE) for university student status among the emerging adult sample, age was treated as a control variable. In addition, prior international experience and language aptitude test experience were considered antecedents to enter into the model (MacNab and Worthley, 2012). Acceptable model fit was determined from conventional consideration of the incremental (CFI, TLI), absolute (SRMR), and parsimonious fit indices (RMSEA), with special attention to CFI and TLI values approached 0.90, SRMR values less than or close to 0.06, and RMSEA values were close to or less than 0.08 (Hooper et al., 2008). Degrees of freedom were also checked with a degrees of freedom calculator (Cortina et al., 2017).

The results of the retained model from SEM are available in Table 3. Iterative model adjustments are available in the repository syntax. A supported model was specified and retained with the following results: Robust χ^2^ (1,493) = 4906.337, *p* = 0.000; RMSEA = 0.042 (95% *CI*:0.040–0.043); CFI = 0.925; TLI = 0.920; SRMR = 0.076. Residuals for one SEIC item (SEIC 1) and *Conscientiousness* and one item for SEIC and *Extraversion* (SEIC 8) were allowed to correlate. In addition, residuals of within-facet pairs for S-SE items were allowed to correlate as in the CFA for S-SE, and residual variance for one item from L-SE (Item 11) were allowed to covary due to item overlap with the other SE scales (“If two foreign people had an English conversation in front of me, I would understand it”).

**TABLE 3:** Results of the supported structural equation model for the relationships between the study variables with emerging adults in Japan (*N* = 1,326).

Variable	SEIC	S-SE	L-SE	FLCA-CA	FLCA-FOF
**Demographics**					
Age	–0.02 (0.01)*	–0.01 (0.01)	–0.05 (0.01)	0.01 (0.01)	0.01 (0.01)
International experience	–0.21 (0.05)***	–0.36 (0.05)***	–0.30 (0.06)***	–0.01 (0.02)	0.01 (0.05)
Language aptitude test experience	–0.35 (0.06)***	–0.64 (0.05)***	–0.77 (0.06)***	–0.02 (0.02)	0.08 (0.05)
**Revised Big Five factor markers^a^**					
Extraversion	0.23 (0.04)***	0.24 (0.04)***	0.16 (0.04)***	0.06 (0.01)***	–0.13 (0.04)***
Neuroticism	–0.17 (0.03)***	–0.12 (0.03)***	–0.04 (0.03)	–0.08 (0.02)***	0.25 (0.03)***
Conscientiousness	0.12 (0.05)*	0.06 (0.04)	0.24 (0.05)***	–0.03 (0.01)*	0.10 (0.05)*
Openness to experience	0.17 (0.04)***	0.12 (0.04)*	0.04 (0.05)	–0.01 (0.01)	0.03 (0.04)

^a^Standardized coefficient results (standard error), controlling for age, prior international travel experience, and language aptitude test experience. Model fit indices, Robust χ^2^ (1,493) = 4906.337; RMSEA (95% CI) = 0.042 (0.040–0.043); CFI/TLI = 0.925/0.920. Residuals for one SEIC item (SEIC 1) and Conscientiousness and one item for SEIC Extraversion (SEIC 8) were allowed to correlate after examination of modification indices.

*p < 0.05, ***p < 0.0001.

Apart for SEIC which had a small, supported correlation (see Table 4) and standardized coefficient for age (β = –0.02, *p* < 0.05), coefficients for all other L2 constructs were not related to age in the model (Table 3). Prior international experience showed strongly negative paths to SEIC (β = –0.21, *p* < 0.0001) and S-SE (β = –0.36, *p* < 0.0001), but was unrelated to FLCA-CA (β = –0.01) and FLCA-FOF (β = 0.01). Prior language test experience showed strongly negative paths to SEIC (β = –0.35, *p* < 0.0001) and S-SE (β = –0.64, *p* < 0.0001), but was unrelated to FLCA-CA (β = –0.02) and FLCA-FOF (β = 0.08).

**TABLE 4:** Means, standard deviations, and correlations with confidence intervals.

Variable	*M*	*SD*	1	2	3	4	5	6	7	8	9
1. Age	25.22	3.08									
2. Extraversion	2.98	1.21	–0.01								
			[–0.06, 0.04]								
3. Neuroticism	4.12	1.07	–0.02	–0.23**							
			[–0.07, 0.04]	[–0.28, –0.18]							
4. Conscientiousness	3.51	1.02	0.02	0.33**	0.08**						
			[–0.03, 0.08]	[0.28, 0.38]	[0.03, 0.14]						
5. Openness to experience	3.56	1.07	–0.07*	0.45**	0.06*	0.41**					
			[–0.12, −0.02]	[0.41, 0.50]	[0.01, 0.12]	[0.36, 0.45]					
6. SEIC	2.46	1.11	–0.09**	0.44**	–0.24**	0.26**	0.34**				
			[–0.14, −0.03]	[0.39, 0.48]	[–0.29, −0.19]	[0.21, 0.31]	[0.29, 0.39]				
7. S-SE	2.38	1.17	–0.06*	0.43**	–0.23**	0.23**	0.32**	0.71**			
			[–0.11, –0.01]	[0.38, 0.47]	[–0.28, –0.18]	[0.18, 0.28]	[0.27, 0.37]	[0.68, 0.73]			
8. L-SE	2.99	1.20	–0.15**	0.33**	–0.13**	0.27**	0.29**	0.55**	0.75**		
			[–0.20, –0.09]	[0.28, 0.38]	[–0.18, –0.08]	[0.22, 0.32]	[0.24, 0.33]	[0.51, 0.59]	[0.73, 0.77]		
9. FLCA-CA	3.07	0.80	–0.02	–0.17**	0.34**	0.07**	0.02	–0.16**	–0.18**	–0.14**	
			[–0.07, 0.03]	[–0.22, –0.12]	[0.29, 0.38]	[0.02, 0.13]	[–0.03, 0.08]	[–0.21, –0.10]	[–0.23, –0.13]	[–0.19, –0.09]	
10. FLCA-FOF	3.17	0.91	0.01	–0.17**	0.35**	0.06*	0.02	–0.21**	–0.24**	–0.20**	0.82**
			[–0.04, 0.06]	[–0.22, –0.12]	[0.31, 0.40]	[0.01, 0.12]	[–0.04, 0.07]	[–0.26, –0.16]	[–0.29, –0.19]	[–0.25, –0.15]	[0.80, 0.84]

*M* and *SD* are used to represent mean and standard deviation, respectively. Values in square brackets indicate the 95% confidence interval for each correlation. The confidence interval is a plausible range of population correlations that could have caused the sample correlation (Cumming, 2014).

*Indicates *p* < 0.05.

**Indicates *p* < 0.01.

Positive standardized coefficients with supported paths (*p*s < 0.0001) were observed for *Extraversion* and SEIC (β = 0.23), L-SE (β = 0.24), and S-SE (β = 0.24), but a negative path was observed for FLCA-FOF (β = –0.13). A similar pattern was observed for *Neuroticism* (*p*s < 0.0001), which showed negative paths to SEIC (β = –0.17), S-SE (β = –0.12) and CA, (β = –0.08), but a positive path for FLCA-FOF (β = 0.24). Contrary to expectations, *Conscientiousness* was strongly related to L-SE (β = 0.24), weakly related to SEIC (β = 0.12), FLCA-CA (β = –0.03), and FLCA-FOF (β = 0.10), and unrelated to S-SE (β = 0.06). Finally, *Openness to Experience* was more strongly supported for a relationship to SEIC (β = 0.17, *p* < 0.0001) than S-SE (β = 0.12, *p* < 0.05), and found to be unrelated for L-SE (β = 0.04), FLCA-CA (β = –0.01) and FLCA-FOF (β = 0.03). These results indicate differential contributions for the patterns of relationships between personality and lower-order L2 constructs for self-efficacy and anxiety domains.

## Discussion

Personality impacts thoughts about the self and others, attitudes, and responses to stressful situations. Individual differences in personality have long been posited to influence language acquisition (Verhoeven and Vermeer, 2002; Piechurska-Kuciel, 2020), language use (Dewaele and Furnham, 1999; Dewaele, 2012), intercultural development while abroad (Ward et al., 2004; Caspi et al., 2006; Hudson and Inkson, 2007; Savicki, 2011; Miao and Harris, 2012), intercultural adjustment (Huff et al., 2014), and related capabilities like cultural intelligence (Ang et al., 2006; Matsumoto and Hwang, 2013). Researchers have lamented the lack of attention given to personality traits in the extant literature (Dewaele, 2012; Wilson et al., 2013). Proximal and distal factors are key sources of validity for nomological networks of constructs rooted in the theory of self-efficacy, or the self-view about the ability to perform, do, or achieve desired actions in a competent manner. As self-efficacy is theorized as a construct relevant to positive development (Shek et al., 2012; Tsang et al., 2012) intercultural development settings (MacNab and Worthley, 2012) and global competence (Organization for Economic Co-operation and Development [OECD], 2019), we aimed to clarify relationships according to their domain specificity.

**FIGURE 1:**
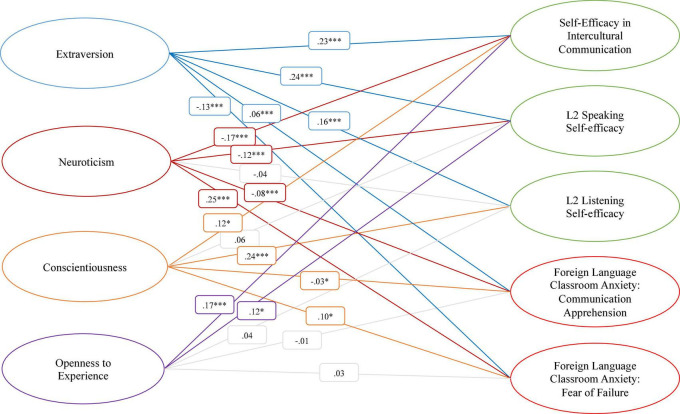
Visual representation of the structural equation model estimating the relations between the Revised Big Five factors, L2 self-efficacy domains, and FLCA factors. Model fit indices, Robust χ*^2^* (1,493) = 4906.337; RMSEA (95% CI) = 0.042 (0.040–0.043); CFI/TLI = 0.925/0.920. Residuals for one SEIC item (SEIC 1) and *Conscientiousness* and one item for SEIC *Extraversion* (SEIC 8) were allowed to correlate after examination of modification indices. *p < 0.05, ^***^p < 0.0001.

This study examined the relationships between personality factors and L2 self-efficacy and foreign language anxiety constructs for emerging adults in Japan with structural equation modeling. Two research questions were explored: (1) How personality traits relate to L2 self-efficacy constructs and (2) FLCA factors in a nationally representative sample of emerging adults in Japan. As depicted in Table 1, mean values for self-efficacy types were relatively low compared to anxiety types, and personality factor scores were highest for *Neuroticism* and *Openness to Experience*. Replicating prior studies with the respective psychometric instruments, reliability coefficients were high for all study variables (Apple, 2011; Toyama and Yamazaki, 2018; Kabir and Sponseller, 2020). As shown in Table 2, measurement models with confirmatory factor analysis generally showed adequate model fit. Intercorrelations for the study variables were convergent for self-efficacy types [*r*(1,324) = 0.55 to 0.75, *p* < 0.01], with correlation coefficients that were notably higher than those observed for university students in Kabir and Sponseller (2020) [L-SE and SEIC, *r*(77) = 0.42, *p* < 0.001; S-SE and SEIC, *r*(77) = 0.28, *p* < 0.001], as seen in Table 4. Negative correlations were observed for the self-efficacy types with FLCAS, suggesting divergent and incremental validity for the self-efficacy types, although the strengths of the relationships were relatively low [*r*(1,324) = –0.14 to –0.24, *p* < 0.01]. Table 3 shows the results of structural equation modeling for age, prior international and test-taking experience, and personality factors as predictors for the L2 self-efficacy and anxiety factors as outcome variables, with coefficients depicted in Figure 1. Evaluation of our proposed hypotheses with the retained model of the geographically diverse sample are discussed in the following sections.

### Personality and second language self-efficacy constructs

For the SEIC, we built upon the results of a former modeling procedure to which the results of the university sample (*n* = 373) showed that SEIC was predicted by Extraversion (β = 0.33, *p* < 0.001) and *Conscientiousness* (β = 0.19, *p* < 0.05), but not *Neuroticism* (β = 0.01) or *Openness to Experience* (β = 0.12) (Supplementary material 1). The former factors were replicated in the larger sample (*n* = 1,326), but the latter were novel for this age group and context of these variables in Japan. Contrary to our expectations in H1 for the nationally representative sample, the results showed that SEIC was predicted by *all* measured personality factors (Table 3), including *Neuroticism* (β = –0.17) and *Openness to Experience* (β = 0.17). These findings for SEIC are similar to previous studies of related constructs of cultural intelligence (Ang et al., 2006), especially for interactional adjustment and *Neuroticism* and *Extraversion* (Huff et al., 2014). Our predictions for H2 were partially supported and resemble findings for *Neuroticism* and *Extraversion* as significant predictors of L2 grit (Teimouri et al., 2020), but unique for *Openness to Experience*.

As clusters of patterns, the supported paths for *Openness to Experience* for SEIC and S-SE are similar to previous findings by Piechurska-Kuciel (2018), which showed evidence for the factor as a significant predictor of willingness to communicate. However, it shows some distinction for L-SE, which lacked this path, suggesting that preferential tendencies for seeking new knowledge and experiences are insufficient for obtaining L-SE among emerging adults in Japan. We believe that the effortful learning required to improve at L2 listening is reflected in the strongest supported path observed for *Conscientiousness* and L-SE, as well as in the weaker path to SEIC, due to its relationship to trait-like self-regulation. This might be due to the commitment to study necessary to obtain high degrees of perceived competence in listening ability (e.g., strategy use and personal control; Graham, 2011) and self-monitoring needed to receptively engage in the reciprocal exchanges of intercultural communication. For S-SE, the strongest patterns of relationships are consistent with previous findings by Oya et al. (2004) who observed relationships between *Extraversion* and *Neuroticism* with global impressions of oral performance. The S-SE findings also mirror those for *Extraversion* and speech production (Dewaele and Furnham, 2000).

While we expected that prior international and language test experience might be positive predictors of self-efficacy types as achievement or committed behavior, to our surprise, the demographic variables of prior international and language experience were strongly *negative* predictors in our study. These results suggest further examination with a composite variable with the other items for prior international experience (e.g., Takeuchi et al., 2005; Ott and Iskhakova, 2019), more comprehensive measures and sophisticated analyses of language achievement and aptitude (e.g., akin to tests of incremental predictive validity for instruments on the self-efficacy of communication in Harris, 2022; related self-concept measures, Leeming and Harris, 2022), or consideration of detailed models with mediators or covariate adjustments for related relationships or pathways (e.g., SES), beginning with graphical causal models for observational data (Rohrer, 2018).

### Personality and factors of foreign language classroom anxiety

In support of our second research question and H3, the antecedents of personality related to FLCA factors in a geographically sample of emerging adults in Japan. Fear of Failure (FOF) was predicted positively by *Neuroticism* (β = 0.25) and *Conscientiousness* (β = 0.10), and negatively by *Extraversion* (β = –0.13), which was mirrored for the Communication Apprehension (CA) factor but with weaker coefficients, and a noted exception of a small but negatively supported path from *Neuroticism* to CA (β = –0.08). The directions of the other paths were mostly theoretically consistent; however, one surprising result was the small, but positively supported path from *Conscientiousness* to FOF, which we expected to be negative in H3. Though small, these results suggest that FLCA is not straightforwardly opposed by self-efficacy beliefs as a typical source of self-enhancement, but rather might represent a balanced form of self-enhancement or possible motivation for *self-improvement* (Joshanloo et al., 2021; see section “Implications for applied educational settings”). Interestingly, the FLCAS factors were unrelated to the demographic predictor variables. While FLCA related to English might be expected to increase after taking English proficiency exams, or some exposure to interactional difficulties due to English while abroad, the lack of supported paths suggests that these were not robust as indicator-coded predictors in our study.

### Implications for applied educational settings

One interpretation of the structural coefficients (Table 3) is that the multivariate relations bear resemblance to the findings of the PISA results on self-efficacy and fear of failure for youth in Japan. The mean scores for all self-efficacy types for emerging adults in the general population from the present study (*M* = 2.46) were lower than Kabir and Sponseller (2020) for university students (*M* = 2.92) and professional educators (*M* = 3.71). This finding is similar to a study of university students from Japan in which self-perceived L2 domain skills in English were low (Chen et al., 2020), aligns with another study showing low levels of English use in terms of speaking skills for those outside of university (Terasawa, 2021), and corresponds generally to other findings for self-efficacy and motivated behavior (e.g., Ueki and Takeuchi, 2013). Considering the higher values for FOF with these lower levels of all L2 self-efficacy types (Table 1), our findings resemble the broader observations of self-efficacy and fear of failure with 15-year-olds in the PISA, 2018 Japan results (Organization for Economic Co-operation and Development [OECD], 2020), representing a possible extension to the foreign language and intercultural skills domains among the developmental group of emerging adults aged 18–29 years old. Our results for FLCA also provide support for the practical approach to teacher adjustments for managing FLA-related fear of failure about mistakes proposed by Toyama and Yamazaki (2018). Together, these implications invite numerous avenues for future theorizing. Among these, reported differences for “balanced” forms *self-enhancement* or the primacy of *self-improvement* (Joshanloo et al., 2021), might be strong candidates for cultural influences on these variables within the context of Japan (e.g., Heine et al., 2000, 2001; Heine and Hamamura, 2007 as cited in Su and Oishi, 2011; Joshanloo et al., 2021). Comparison of self-efficacy types with wellbeing measures might reveal these relationships, as indicated in a conceptual analysis of the relationships between positive psychology and foreign/second language acquisition (e.g., Wang et al., 2021), and implied in work applying the framework of self-determination theory (e.g., McEown and Oga-Baldwin, 2019) and other comprehensive treatments that extend and integrate the perspective of teaching to the psychology of language learning (Gregersen and Mercer, 2022).

While empirical and implementation studies will need to be conducted before drawing strong inferences about pedagogical mechanisms of change, our findings suggest that matching domains to approaches that offer incremental, concrete, and constructive feedback might be useful for learners in order to provide self-improvement opportunities. Thus, the chief implication of our findings to applied educational settings would be that trait coherence for intercultural interactions (for SEIC), connected speech (for L-SE), and oral performance with feedback (for S-SE) might be routes to leverage tendencies in personality toward enhancing L2 self-efficacy beliefs. The pattern of SEIC relating to all measured personality factors implies that a focus on improving intercultural communication might be suitable to achieve a wide appeal to student or emerging adult dispositions (e.g., pragmatic awareness or competence in an L2), or selection and socialization effects from learning experiences (e.g., Nissen et al., 2022) though detailed implementation research will be necessary.

### Limitations and future directions

A strength of our study is the focus on this age group in a nationally representative sample, which provides broader context for patterns among these variables beyond student populations. In addition, strong measurement invariance was indicated for the factor models of the RB5-J, SEIC and S-SE, suggesting generalizability with previous findings (Apple, 2011; Kabir and Sponseller, 2020). However, one of the major limitations is the lack of a measurement model that includes *Agreeableness* as a factor, which might limit cross-cultural generalizability and applications to practical settings (e.g., *Agreeableness* connections to pair work, Karlin and Karlin, 2017). While we replicated the factor model by Apple (2011), suggesting a precise measurement protocol, research on instruments that include the full complement of Big Five factors in Japan have recently been conducted (Toyomoto et al., 2022). Future research might consider these tools or other personality frameworks that consider other culturally shaped factors (i.e., Honesty-Humility in HEXACO; Wakabayashi, 2014), or trait clusters of them (Piechurska-Kuciel, 2020) as joint effects (e.g., using techniques implemented by Schlaegel et al., 2021).

In addition, caution is advised for strong interpretations of our results as we are unable to make causal claims about relationships due to our limited, observational study design. A longitudinal observation for the measured self-efficacy types as study variables is also still necessary and planned for future research. Another limitation is the overlap between tests, which is evident from the modification indices and freed parameters needed to estimate the model (e.g., L-SE item 11; item-pairs for S-SE). While internal validity is apparent, external validity evidence for the factor models of L-SE and S-SE are still needed to overcome potential bias, threats to validity, and construct separation (i.e., to evaluate the relatively high intercorrelations between L-SE and S-SE). Addressing these points and the demographic results, future research might include prior language experience variables with the LEAP-Q (Kaushanskaya et al., 2020), predictive validity to lexical retention among advanced learners in Japan (e.g., Nakata et al., 2020), convergence with tools for communicative SE (Harris, 2022) or English-related SE and self-regulated learning strategies (Kim et al., 2015). For SEIC, empirical relationships to other strong theories like cultural intelligence, positive youth development, self-determination theory, or flourishing constructs might further bolster or scrutinize the fit of the construct within relevant nomological networks (Ang and Van Dyne, 2008; Van Dyne et al., 2008; MacNab and Worthley, 2012; Rockstuhl and Van Dyne, 2018). Qualitative and mixed-methods study designs with varied methodologies (e.g., Gkonou and Oxford, 2016; Irie et al., 2018) will also provide illustration, context, complementarity, and triangulation for our study variables, which are limited to self-report in this study.

## Conclusion

This study provided detailed evidence of structural relations between personality factors and domains of L2 self-efficacy and anxiety constructs for emerging adults in Japan from an online survey of 1,326 participants. We observed partial support for our hypothesized relationships. The structural coefficients with the strongest magnitude emphasize the role of *Extraversion* and *Neuroticism* in L2 language skill domain-related self-efficacy, intercultural communication, and classroom anxiety. The results suggest domain-specific patterns for *Openness to Experience*, and the opposing signs for *Conscientiousness* suggest *self-improvement*, rather than *self-enhancement*, might be reflected in L2 self-efficacy beliefs for emerging adults in Japan. Future research is needed to determine the contributions of *Agreeableness* or factors from other measurement frameworks of personality. Practitioners might consider SEIC-related skills as a domain relevant to at least four personality types, which might allow for broad applications to coursework that incorporates global competence (e.g., pragmatic awareness and intercultural communication).

## Data availability statement

The datasets presented in this study can be found in online repositories. The names of the repository/repositories and accession number(s) can be found below: Open Science Framework: https://osf.io/9yx7a/?view_only=32946bdef6d645f0ac302fde5ab2cae1.

## Ethics statement

The studies involving human participants were reviewed and approved by the Ethics Review Board, Graduate School of Humanities and Social Sciences, Hiroshima University. The patients/participants provided their written informed consent to participate in this study.

## Author contributions

RK served as the primary author of the manuscript and contributed to conceptualization, investigation, methodology, software, formal analysis, original draft writing, funding acquisition, data curation, visualization, revisions, project administration, and supervision. BK contributed to resources, investigation, original draft writing, and revisions. MK provided literature review and revisions. AS contributed to funding acquisition, data curation, formal analysis, validation, project administration, revisions, and supervision. All authors contributed to the article and approved the submitted version.
