# Supplementary material for: Modeling personality antecedents and second language self-efficacy constructs with emerging adults in Japan: Domain-specific matching for assessing global competence in applied contexts
Source: Front Psychol. 2022 Dec 15;13:1032573. doi: 10.3389/fpsyg.2022.1032573 (PMC9799980; doi:10.3389/fpsyg.2022.1032573)
Supplement: Supplementary file 1 [file Data_Sheet_1.docx]

Modeling personality antecedents and L2 self-efficacy constructs with emerging adults in Japan: Domain-specific matching for assessing global competence in applied contexts

***Supplementary Material 1***

In this section, we report descriptive statistics (*M, SD*), model fit indices, and results for the sample of 373 university students whose self-efficacy in intercultural communication (SEIC) and big five personality (RB5) data was collected from three university sites in Japan. The sample was comprised of undergraduate students from Hiroshima University, Osaka Jogakuin College, and Osaka International University. The study was reviewed and approved according to the ethical research protocols from all three institutions. The researchers underwent multiple data collection techniques (purposive and convenient) sampling approaches for recruitment. Internal consistency of the measures was assessed with Cronbach’s alpha and McDonald’s omega coefficients (Dunn, Baguley, & Brunsden, 2014). Following quantitative reporting standards (Appelbaum et al., 2018), model fit for CFA and SEM was assessed in terms of Chi-square tests, Standardized Root Mean Square Residual (SRMR; acceptable < .08), the Tucker Lewis Index (TLI; acceptable > .90), the Root Mean Square Error of Approximation (RMSEA; acceptable < .08), and the Comparative Fit Index (CFI; acceptable > .90, Hooper, Coughlan, and Mullen, 2008).

**References:**

Appelbaum, M., Cooper, H., Kline, R. B., Mayo-Wilson, E., Nezu, A. M., and Rao, S. M.

(2018). Journal article reporting standards for quantitative research in psychology: the APA publications and communications board task force report. *Am. Psychol.* 73, 3–25. doi: 10.1037/amp0000191

Brown, T. A. (2015). *Confirmatory Factor Analysis for Applied Research*. Guilford

Publications.

Dunn, T. J., Baguley, T., and Brunsden, V. (2014). From alpha to omega: a practical solution to

the pervasive problem of internal consistency estimation. *Br. J. Psychol.* 105, 399–412. doi: 10.1111/bjop.12046

Hooper, D., Coughlan, J., & Mullen, M. R. (2008). Structural equation modelling: Guidelines

for determining model fit. *Electronic Journal of Business Research Methods*, *6*(1), pp 53-60.

R Core Team (2020). R: A language and environment for statistical computing. R Foundation

for Statistical Computing, Vienna, Austria. Available at: https:// www.R-project.org/

Rosseel, Y., Oberski, D., Byrnes, J., Vanbrabant, L., Savalei, V., Merkle, E., et al.

(2020). Package ‘lavaan’.

**Table 1.** *Descriptive statistics of the study variables with university students in Japan (n = 373)*

| Study variable | University students in Japan |
| --- | --- |
| Revised Big Factor Markers (*M*, *SD*)  Emotional Stability (4 items; *α* = .78, *ω* = .79 [.74, .82])  Conscientiousness (3 items; *α* = .74, *ω* = .74 [.67, .79])  Extraversion (4 items; *α* = .83, *ω* = .83 [.80, .86])  Openness to Experience (3 items; *α* = .75, *ω* = .79 [.74, .82]) | 3.97 (1.00) 3.45 (0.95) 3.95 (1.03) 3.97 (0.99) |
| Self-Efficacy in Intercultural Communication (*M*, *SD*)  SEIC (8 items; *α* = .88, *ω* = .88 [.86, .90]) | 2.94 (0.87) |

*Note. M* and *SD* are used to represent mean and standard deviation, respectively. α and ω are used to represent Cronbach’s alpha and McDonald’s omega coefficients, respectively. Response categories for the Revised Big Five Factors and SEIC ranged from 1-6.

**Table 2.** *Model comparison of factor structures and psychometric properties for the study instruments.*

| Model | | *df* | Robust Minimum Function Test Statistic *(χ^2^)* | *χ^2^ p*-value | Robust CFI | Robust TLI | SRMR | Robust RMSEA (CI) |
| --- | --- | --- | --- | --- | --- | --- | --- | --- |
| Self-Efficacy in Intercultural Communication | 1-factor model | 20 | 86.760 | 0.000 | .927 | .898 | .046 | .118 (.098-.139) |
|  |  |  |  |  |  |  |  |  |
| Revised Big Five Factor Markers | 1-factor model | 629 | 3197.571 | 0.000 | .413 | .379 | .128 | .117 (.113-.121) |
|  | 5-factor model | 550 | 1778.830 | 0.000 | .706 | .682 | .094 | .086 (.082, .091) |
|  | 4-factor model | 71 | 145.803 | 0.000 | .947 | .932 | .057 | .059 (.045-.072) |

*Note.* The values for the test and fit statistics are reported for the results of the model with the robust maximum likelihood estimator. CFI = Comparative Fit Index. TLI = Tucker-Lewis Index. SRMR = Standardized Root Mean Square Residual. RMSEA = Root Mean Square Error of Approximation.

**Table 3.** *Results of the supported structural equation model for relationships between self-efficacy in intercultural communication and factors from the revised Big Five model for undergraduate students from three universities in Japan (n = 373).*

| *Variable* | *Self-Efficacy in Intercultural Communication* |
| --- | --- |
| Demographics |  |
| Gender | -.01 (.17) |
| Big Five Factor Markers ^a^ |  |
| Extraversion | .33 (.08)** |
| Emotional Stability | .01 (.06) |
| Conscientiousness | .19 (.09)* |
| Openness to Experience | .12 (.07) |

*Note.* Model Fit Indices, Robust *χ^2^* (*df*) = 398.665 (218); Robust RMSEA (95% CI) = .056 (.048-.065); Robust CFI/TLI = .919/.906; ^a^ Standardized coefficient results, controlling for gender. * *p* < 0.05 ** *p* < 0.001
