# Supplementary material for: Modeling personality antecedents and second language self-efficacy constructs with emerging adults in Japan: Domain-specific matching for assessing global competence in applied contexts
Source: Front Psychol. 2022 Dec 15;13:1032573. doi: 10.3389/fpsyg.2022.1032573 (PMC9799980; doi:10.3389/fpsyg.2022.1032573)
Supplement: Supplementary file 2 [file Data_Sheet_2.docx]

Modeling personality antecedents and L2 self-efficacy constructs with emerging adults in Japan: Domain-specific matching for assessing global competence in applied contexts

***Supplementary Material 2***

**REVISED BIG FIVE FACTOR ITEMS**

**Instructions:**

The following questions are about your personality. Please select the response that best describes you.

あなたの性格について伺います。以下の項目について、最も当てはまるものを選択してください。

**Response Scale**

1 = Completely disagree 全くそう思わない

2 = Mostly disagree そう思わない

3 = Disagree a little あまりそう思わない

4 = Agree a little 少しそう思う

5 = Mostly agree そう思う

6 = Completely agree 非常にそう思う

*Extraversion (EX)*

EX1 Am the life of the party

盛り上げ役である。

EX2 Like to talk to a lot of people

いろいろな人と話すのが好きだ。

EX3 Start conversations

人に自分から話し掛ける。

EX4 Like to draw attention to myself

人の注意を引くのが好きだ。

EX5 Like to be the center of attention

注目の的になるのが好きだ。

EX6 Talk a lot

よくしゃべる。

EX7 Feel at ease in a large group of people

たくさんの人の中にいると落ち着く。

EX8 Am skilled at dealing with people

人と関わるのが上手だ。

*Agreeableness (AG)*

AG1 Inquire about others’ well-being

周りの人の様子を尋ねる。

AG2 Feel concern for others

周りの人への関心がある。

AG3 Am interested in other people’s problems

周りの人の問題に興味がある。

AG4 Take time out for others

周りの人のために時間を割く。

AG5 Am on good terms with nearly everyone

ほぼ誰とでも仲良くなれる。

AG6 Make people feel at ease

周りの人を安心させる。

AG7 Have a good word for everyone

誰についても良いことを言える。

AG8 Know how to cheer people up

周りの人を元気づけることができる。

*Openness (OP)*

OP1 Have a vivid imagination

想像力が豊かだ。

OP2 Am full of ideas

発想が豊かだ。

OP3 Have a rich vocabulary in my native language

日本語の語彙力が豊富だ。

OP4 Am quick to understand things

ものわかりが良い。

OP5 Love to read challenging materials

難しい書物を読むのが好きだ。

OP6 Love to think of new ways to do things

新しいやり方を試すのが好きだ。

OP7 Can handle a lot of information

たくさんの情報に対応できる。

*Conscientiousness (CO)*

CO1 Am prepared

用意周到である。

CO2 Put things back in their proper places

身の回りを整理整頓する。

CO3 Like rules

規則正しいのが好きだ。

CO4 Follow a daily schedule

毎日の予定に従う。

CO5 Do things according to a plan

計画通りにする。

CO6 Like to clean up things

片付けるのが好きだ。

CO7 Continue my work until it’s perfect

物事を完璧にする。

*Emotional Stability (ES)*

ES1 Often feel blue

落ち込むことが多い。

ES2 Get stressed out easily

すぐにストレスがたまる。

ES3 Worry about things

心配性である。

ES4 Have frequent mood swings

気分が変わりやすい。

ES5 Panic easily

慌てやすい。

ES6 Feel threatened easily

びくびくしやすい。

ES7 Get angry easily

怒りやすい。

**HOW TO CITE:**

THE JAPANESE TRANSLATION & VALIDATION

Apple, M. T. (2011). *The Big Five personality traits and foreign language speaking confidence among Japanese EFL students* [Unpublished doctoral dissertation]. Temple University.

**SELF-EFFICACY IN INTERCULTURAL COMMUNICATION (SEIC) ITEMS**

**Instructions:**

How well can you handle the following situations in English-speaking countries? Please choose the response that best describes your ability.

以下の項目について、英語を母国語とする国でどのくらい上手にできますか。最も当てはまるものを選択してください。

**Response Scale:**

1 = Cannot do it well at all 全く上手でない

2 = Cannot do it well 上手でない

3 = Cannot do it very well あまり上手でない

4 = Can do it somewhat well 少し上手だ

5 = Can do it very well 上手だ

6 = Can do it very easily 非常に上手だ

**Items:**

SEIC1 How well can you think possible outcomes through before you speak?

話をする前に起こりうる結果を十分に考える。

SEIC2 How well are you able to adapt to an interaction in which the topic changes from familiar to unfamiliar territory?

トピックが馴染みのあるものからそうでないものに移行した時, 会話についていく。

SEIC3 How well can you communicate with people who are in positions of authority?

自分より高い地位にある人達とコミュニケーションを図る。

SEIC4 When in a face to face conversation, how well can you gauge what another person wants you to communicate?

面と向かって話をする時, 相手があなたと話したい事が何かを判断する。

SEIC5 How well can you recognize subtle shades of meaning in an interaction?

対話の中で, 曖昧な意味を理解する。

SEIC6 How well can you communicate in impromptu situations?

準備のできない状況（即興）で, コミュニケーションをとる。

SEIC7 How well can you build consensus when you communicate?

意見交換の際, 意見をまとめ一致に導く。

SEIC8 How well can you communicate with people you don’t like?

嫌いな人とコミュニケーションを図る 。

**HOW TO CITE:**

THE ORIGINAL AUTHORS MUST BE CITED:

Peterson, J. C., Milstein, T., Chen, Y.-W., & Nakazawa, M. (2011). Self-efficacy in intercultural communication: The development and validation of a sojourners’ scale. *Journal of International and Intercultural Communication*, *4*(4), 290–309. <https://doi.org/10.1080/17513057.2011.602476>

WHEN USING THE SHORT FORM JAPANESE ITEMS:

Kabir, R. S., & Sponseller, A. C. (2020). Interacting with competence: A validation study of the self-efficacy in intercultural communication scale-short form. *Frontiers in Psychology*, *11*. https://doi.org/10.3389/fpsyg.2020.02086

**LISTENING SELF-EFFICACY SCALE**

**INSTRUCTIONS**:

The following questions are about listening skills. For each of the following items, please select the one that best applies.

リスニング技能に関する質問です。以下の項目について、最も当てはまるものを選択してください。

**RESPONSE SCALE**:

1 = Can’t do it at all 全くできない

2 = Probably can’t do it きっとできない

3 = Maybe can’t do it たぶんできない

4 = Maybe can do it たぶんできる

5 = Probably can do it きっとできる

6 = Definitely can do it 確実にできる

**ITEMS:**

LSE1 If I heard an English conversation at the level of a Junior High School textbook, I would understand it.

中学校の教科書に書いてあるレベルの英会話を聞いて理解する。

LSE2 If a Japanese teacher spoke to me slowly in English I would understand it.

日本人の先生から英語でゆっくり話しかけられて理解する。

LSE3 If a foreign teacher speaks slowly in English to me I would understand it.

外国人の先生から英語でゆっくり話しかけられて理解する。

LSE4 If a Japanese person gave a self-introduction in English I would understand it.

日本人の英語の自己紹介したら理解する。

LSE5 If I heard an English conversation at the level of a Senior High School textbook, I would understand it.

高校の教科書に書いてあるレベルの英会話を聞いて理解する。

LSE6 If I was given class directions in English I would understand them.

授業の行動を英語で指示されて、理解する。

LSE7 If a foreigner gave a self-introduction in English I would understand it.

外国人の英語の自己紹介を理解する。

LSE8 If words I recognize when reading were read to me with native pronunciation I would understand them.

読んだらわかる英単語をネイティブ発音で聞いて理解する。

LSE9 If I heard an English conversation at the level of the Center Test or TOEIC, I would understand it.

センター試験やTOEICに出てくるレベルの会話を聞いて理解する。

LSE10 If I watch an English movie without subtitles, I would understand the contents.

英語の映画を見て、字幕なしで内容を理解する。

LSE11 If two foreign people had an English conversation in front of me I would understand it.

目の前で2人の外国人が英語で会話していて、内容を理解する。

LSE12 If I watched the news in English I would understand it.

ニュースを英語で見て理解する。

LSE13 If I listened to an American president’s speech I would understand it.

アメリカの大統領のスピーチを聞いて理解する。

LSE14 If I was spoken to in English on the phone I would understand it.

電話で英語を話されて理解する。

**HOW TO CITE:**

Kramer, B. L., & Denison, C. (2016). *Accurately measuring L2 listening self-efficacy* [Presentation]. JALT 2016 42nd Annual International Conference on Language Teaching and Learning, and Educational Materials Exhibition, Nagoya.

**SPEAKING SELF-EFFICACY SCALE**

**Instructions:**

In the following situations, please select the one that best describes the degree to which you can communicate.

以下の場面で、どの程度コミュニケーションをとれるか、最も当てはまるものを選択してください。

**Response Scale:**

1 = Cannot do it at all 全くできない

2 = Cannot do it できない

3 = Somewhat unable to do it どちらかというとできない

4 = Somewhat able to do it どちらかというとできる

5 = Can do it できる

6 = Definitely can do it 確実にできる

**ITEMS:**

SSE1 Respond to greetings from international students on campus in English.

キャンパス内で外国人留学生からの挨拶に英語で返答する。

SSE2 Guide international students to the school office in English on campus.

キャンパス内で外国人留学生を学校の事務所に英語で案内する。

SSE3 Guide international students to the library on campus in English.

キャンパス内で外国人留学生を図書館へ英語で案内する。

SSE4 Order food at a fast food restaurant in English while traveling abroad.

海外旅行中にファーストフード店で料理を英語で注文する。

SSE5 Ordering food in a restaurant in English while traveling abroad

海外旅行中にレストランで料理を英語で注文する。

SSE6 Purchase a train ticket in English while traveling abroad.

海外旅行中に電車の切符を英語で購入する。

SSE7 Book a double room with bathroom in English when traveling abroad on your own

自分自身で海外旅行をするとき、バスルーム付きの2人部屋を英語で予約する。

SSE8 Make a reservation for a double room with bathroom for a trip abroad in English over the phone.

電話で、海外旅行のバスルーム付きの2人部屋を英語で予約する。

SSE9 Help a foreign tourist who is lost near your house in English.

あなたの家の近くで迷っている外国人観光客を英語で助ける。

SSE10 Explain how to cook a typical Japanese dish in English.

代表的な日本料理を作る方法を英語で説明する。

SSE11 Ask for help in English if you get lost while traveling abroad.

海外旅行中、迷子になった際、英語で助けを求める。

SSE12 Ask a pharmacist in a foreign pharmacy if they have headache medicine in English

外国の薬局で薬剤師に頭痛薬があるかを英語で尋ねる。

SSE13 Ask a pharmacist about the side effects of headache medicine in a foreign pharmacy in English.

外国の薬局で頭痛薬の副作用について薬剤師に英語で尋ねる。

SSE14 In a restaurant, explain that you are allergic to eggs and ask the waiter in English to ask the chef if the dish you want to order contains eggs.

レストランで、卵アレルギーであることを説明し、ウェイターにあなたが注文したい料理卵が含まれているかシェフに聞くように英語でお願いする。

SSE15 apologize to your English teacher in English for being late for class.

英語の先生に授業に遅刻したことを英語で謝る。

SSE16 Explain to your teacher that you missed last week's class and ask her in English what you missed.

あなたが先週の授業を欠席したことを先生に説明し、また欠席した授業内容について英語で聞く。

SSE17 Explain to your English teacher in English that you were late for class because of the train delay.

授業に遅れたことが電車の遅延のためであることを英語の先生に英語で説明する。

SSE18 Explain to the English teacher in English that you were late for class because of a train delay, but you could not get a delay certificate at the station.

授業に遅れたのは電車の遅延のためであるが、その駅で遅延証明をもらうことができなかったことを英語で説明する。

SSE19 Explain in English that you are late for class because you cannot get on the bus because more people than usual are using the bus due to the rain and the bus is full. Explain this in English.

雨のせいで普段より多くの人々がバスを使い、バスが満員になったため、あなたは乗車することができなくて授業に遅れます。そのことを英語で説明する。

SSE20 As a result, you are late for class and you feel that it is unfair that you are considered late. Assert this in English.

その結果授業に遅刻したあなたは、それが遅刻と見なされるが不公平だと感じます。これを英語で主張する。

**HOW TO CITE:**

Hicks, D., & McLean, S. (2014). *The validation of WTC measurement instruments* [Presentation]. JALT 2014 40th Annual International Conference on Language Teaching and Learning, and Educational Materials Exhibition, Tsukuba.
